# Supplementary material for: The characterization of variable new antigen receptors targeting FAP isolated from a novel immunized library
Source: Commun Biol. 2025 Aug 13;8:1210. doi: 10.1038/s42003-025-08610-x (PMC12350670; doi:10.1038/s42003-025-08610-x)
Supplement: Supplementary file 1 — Supplementary Information [file 42003_2025_8610_MOESM1_ESM.pdf]

Supplemental Information

The characterization of variable new antigen receptors targeting FAP isolated from a novel immunized library

Gihan S. Gunaratne[1*], Joseph P. Gallant[1*], Kendahl L. Ott[1,2*], Payson L. Broome[1], Sasha Celada[1,3], Jayden L. West[1,2], Jason C. Mixdorf[4], Eduardo Aluicio-Sarduy[4,5], Jonathan W. Engle[4,5], Eszter Boros[6], Labros Meimetis[5], Joshua M. Lang[7,8], Shuang G. Zhao[8,9], Reinier Hernandez[4,5], David Kosoff[7,8,10], Aaron M. LeBeau[1,5,8,‡]

[1]Department of Pathology and Laboratory Medicine, University of Wisconsin School of Medicine and Public Health, Madison, WI 53705, USA

[2]Molecular and Cellular Pharmacology Program, University of Wisconsin School of Medicine and Public Health, Madison, WI, 53705, USA

[3]Cellular and Molecular Pathology Program, University of Wisconsin School of Medicine and Public Health, Madison, WI, 53705, USA

[4]Department of Medical Physics, University of Wisconsin School of Medicine and Public Health, Madison, WI 53705, USA

[5]Department of Radiology, University of Wisconsin School of Medicine and Public Health, Madison, WI 53705, USA

[6]Department of Chemistry, University of Wisconsin-Madison, Madison, WI 53706, USA

[7]Department of Medicine, University of Wisconsin School of Medicine and Public Health, Madison, WI, 53705, USA

[8]University of Wisconsin Carbone Cancer Center, University of Wisconsin School of Medicine and Public Health, Madison, WI 53705, USA

[9]Department of Human Oncology, University of Wisconsin School of Medicine and Public Health, Madison, WI, 53705, USA

[10]William S Middleton Memorial Veterans' Hospital, Madison, WI, 53705, USA

*These authors contributed equally to this work

‡Corresponding Author:
Aaron M. LeBeau, PhD
Department of Pathology and Laboratory Medicine
Department of Radiology
University of Wisconsin Carbone Cancer Center
University of Wisconsin School of Medicine and Public Health
Madison, Wisconsin 53705, United States
Phone:608-262-5586; Email: aaron.lebeau@wisc.edu

Supplemental Figure 1

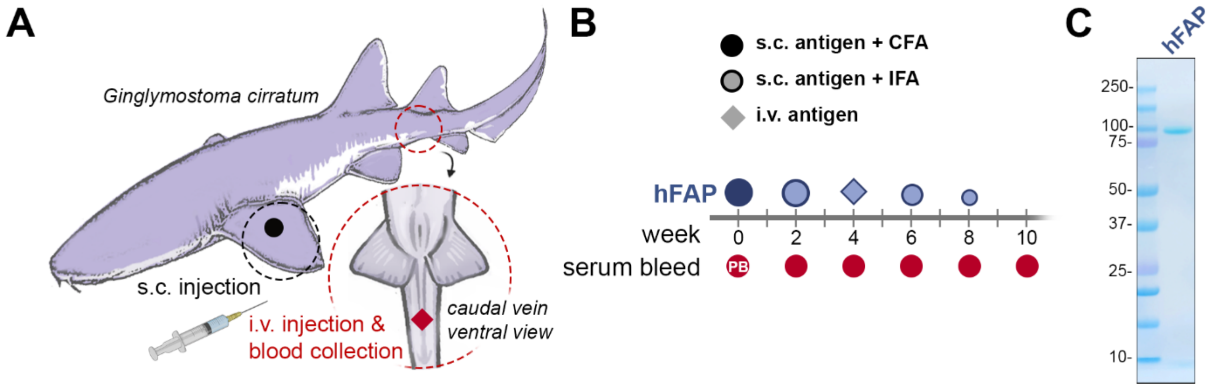

Supplemental Figure 1, Immunization strategy of a live nurse shark against human FAP. A) Schematic of sites used for subcutaneous (s.c.) or intravenous (i.v.) delivery of immunogens and blood collection. The image was made using BioRender (University of Wisconsin license). **B)** Illustration of the time course, injection sites, adjuvants used, and blood sample collection schedule throughout the FAP immunization program. **C)** SDS-PAGE and Coomassie staining of purified recombinant human FAP (hFAP) protein used for immunization.

Supplemental Figure 2

clone ID	clone frequency (%)	FW1	CDR1	FW2	HV2	FW3a	HV4	FW3b	CDR3	FW4
H17	1.2	ARVDQTPQTITKETGESLTINCVLR	DSNCALSS	TYWYRK	KSGSTNKESISKG	GRYVETV	NSGSK	SFSLRINDLTVEDSGTYRCKV	VYNWSEYDCGNSRFNYDV	YGDGTAVTVN
H12	1.2	ARVDQTPQTITKETGESLTINCVLR	DRKCALSS	TYWYRK	KSGSTNEESIKKG	GRYVETV	NSGSK	SFSLRINDLTVEDSGTYRCNV	LMSWYGYPNEGLECWSDV	YGGGTVVTVN
H15	1.2	ARVDQTPQTITKETGESLTINCVLR	DRKCALSS	TYWYRK	KSGSTNEESIKKG	GRYVETV	NSGSK	SFSLRINDLTVEDSGTYRCNV	LMSWYGYPNEGLECWSDV	YGDGTVVTVN
H13	1.2	ARVDQTPQTITKETGESLTINCVL-	DSNCALSS	TYWYRK	KSGSTNEESISKG	GRYVETV	NSGSK	SFSLRINDLTVENSGTYRCNV	YVAGM----SPCLSWGDV	YGGGTVVTVN
H16	1.2	ARVDQTPQTITKETGESLTINCVLR	DSNCALSS	TYWYRK	KSGSTNEESISKG	GRYVETV	NSGSK	SFSLRINDLTVEDSGMYRCNV	YVAGM----SPCLNWGDV	YGGGTVVTVN
H2	4.9	ARVDQTPQTITKATGESLTINCVLR	DSNCALSS	TYWYRK	KSGSTNEESISKG	GRYVETV	ISGSK	SFSLRINDLTVEDSGTYRCNV	YVAGM----SPCLSWGDV	YGGGTVVTVN
H11	1.2	ARVDQTPQTITKATGESLTINCVLR	DSNCALSS	TYWYRK	KSGSTNEESISKG	GRYVETV	ISGSK	SFSLRINDLTVEDSGTYRCNV	YVAGM----SPCLSWGDV	YGGGTAVTVN
H14	1.2	ARVDQTPQTITKETGESLTINCVLR	DSNCALSS	TYWYRK	KSGSTNEESISKG	GRYVETV	NSGSK	SFSLRINDLTVEDSGTYRCNV	YVAGM----SPCLNWGDV	YGDGTAVTVN
H10	2.4	ARVDQTPQTITKETGESLTINCVLR	DSNCALSS	TYWYRK	KSGSTNEESISKG	GRYVETV	NRGSK	SFSLRINDLTVEDSGTYRCNV	YVAGM----SPCLSWGDV	YGGGTVVTVN
H9	2.4	ARVDQTPQTITKATGESLTINCVLR	DSNCALSS	TYWYRK	KSGSTNEESISKG	GRYVETV	ISGSK	SFSLRINDLTVEDSGTYRCNV	YVAGM----SPCLSWGDV	YGDGTAVTVN
H8	1.2	ARVDQTPQTITKETGESLTINCVLR	DSNCALSS	TYWYRK	KSGSTNEESISKG	GRYVETV	ISGSK	SFSLRINDLTVEDSGTYRCNV	YVAGM----SPCLSWGDV	YGDGTAVTVN
H7	7.3	ARVDQTPQTITKETGESLTINCVLR	DSNCALSS	TYWYRK	KSGSTNEESISKG	GRYVETV	NRGSK	SFSLRINDLTVEDSGTYRCNV	YVAGM----SPCLSWGDV	YGGGTAVTVN
H6	6.1	ARVDQTPQTITKETGESLTINCVLR	DSNCALSS	TYWYRK	KSGSTNEESISKG	GRYVETV	NSGSK	SFSLRINDLTVEDSGTYRCNV	YVAGM----SPCLSWGDV	YGDGTVVTVN
H3	2.4	ARVDQTPQTITKETGESLTINCVLR	DSNCALSS	TYWYRK	KSGSTNEESISKG	GRYVETV	NRGSK	SFSLRINDLTVEDSGTYRCNV	YVAGM----SPCLSWGDV	YGDGTAVTVN
H1	12.2	ARVDQTPQTITKETGESLTINCVLR	DSNCALSS	TYWYRK	KSGSTNEESISKG	GRYVETV	NSGSK	SFSLRINDLTVEDSGTYRCNV	YVAGM----SPCLSWGDV	YGGGTAVTVN
H4	19.5	ARVDQTPQTITKETGESLTINCVLR	DSNCALSS	TYWYRK	KSGSTNEESISKG	GRYVETV	NSGSK	SFSLRINDLTVEDSGTYRCNV	YVAGM----SPCLSWGDV	YGDGTAVTVN
H5	32.9	ARVDQTPQTITKETGESLTINCVLR	DSNCALSS	TYWYRK	KSGSTNEESISKG	GRYVETV	NSGSK	SFSLRINDLTVEDSGTYRCNV	YVAGM----SPCLSWGDV	YGDGTVVTVN

NGS 2405

ARVDQTPQTITKETGESLTINCVLRDSNCALSFTYWYRKKSGSTNEESISKGGRYVETVNSGSKSFSLRINDLTVEDSGTY
RCNVYVGGGCPHWIDVYGDGTAVTVN

Supplemental Figure 2, amino acid sequences of anti-FAP VNARs identified by biopanning and NGS2405. Phagemids encoding anti-FAP VNARs were sequenced by Sanger sequencing, translated amino acid sequences are shown, along with their associated clone ID and the frequency of repeat sequences found among hit clones. Complementarity determining regions (CDR1, CDR3) and hypervariable loops (HV2, HV4) are depicted with black or gray shading, respectively.

Supplemental Figure 3

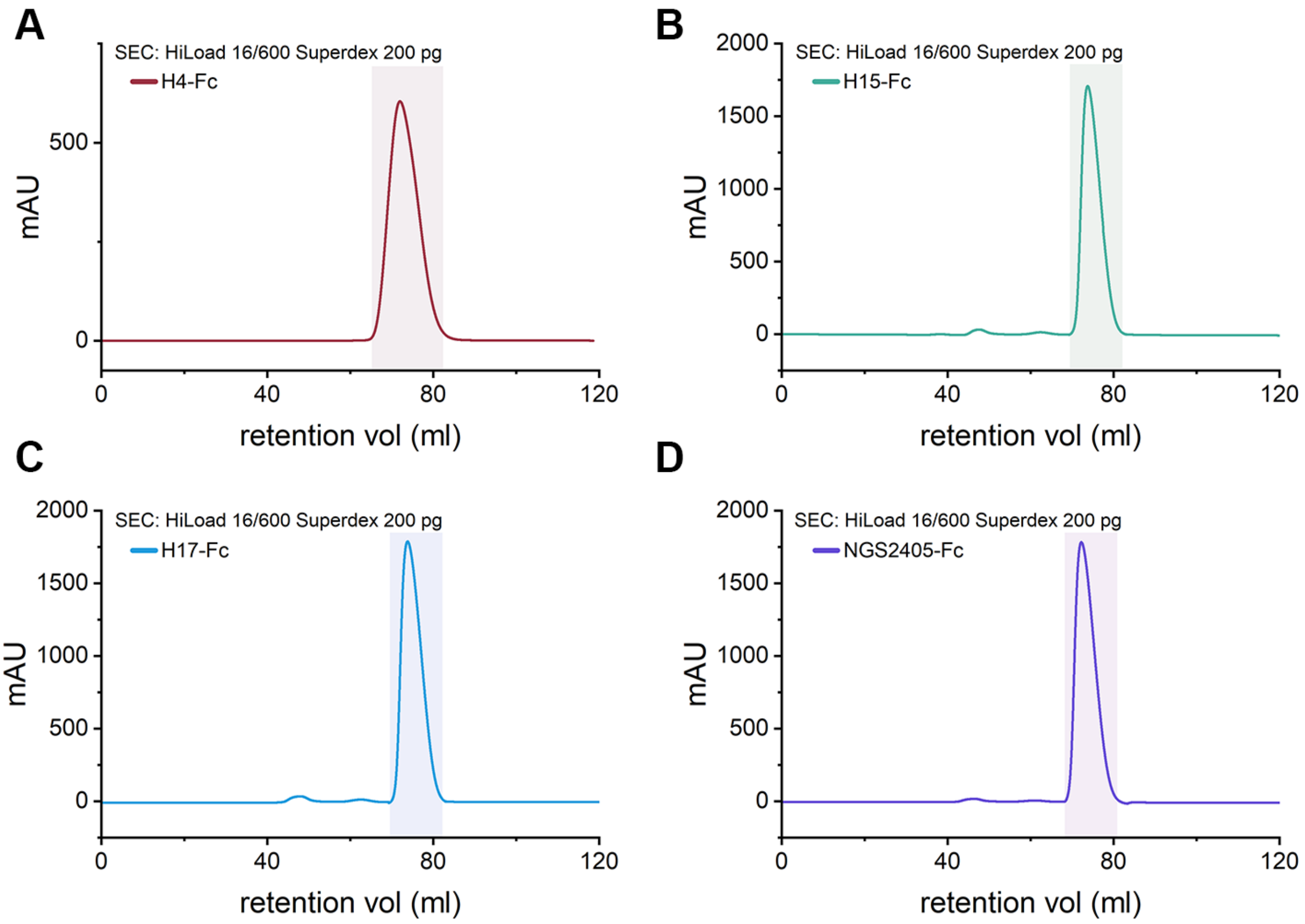

Supplemental Figure 3, Size exclusion chromatography of VNAR-Fc constructs. Anti-FAP VNAR-Fc constructs were purified by protein A affinity chromatography, size exclusion chromatography. Chromatograms of SEC of H4-Fc (A), H15-Fc (B), H17-Fc (C) and NGS2405-Fc (D) are shown.

Supplemental Figure 4.

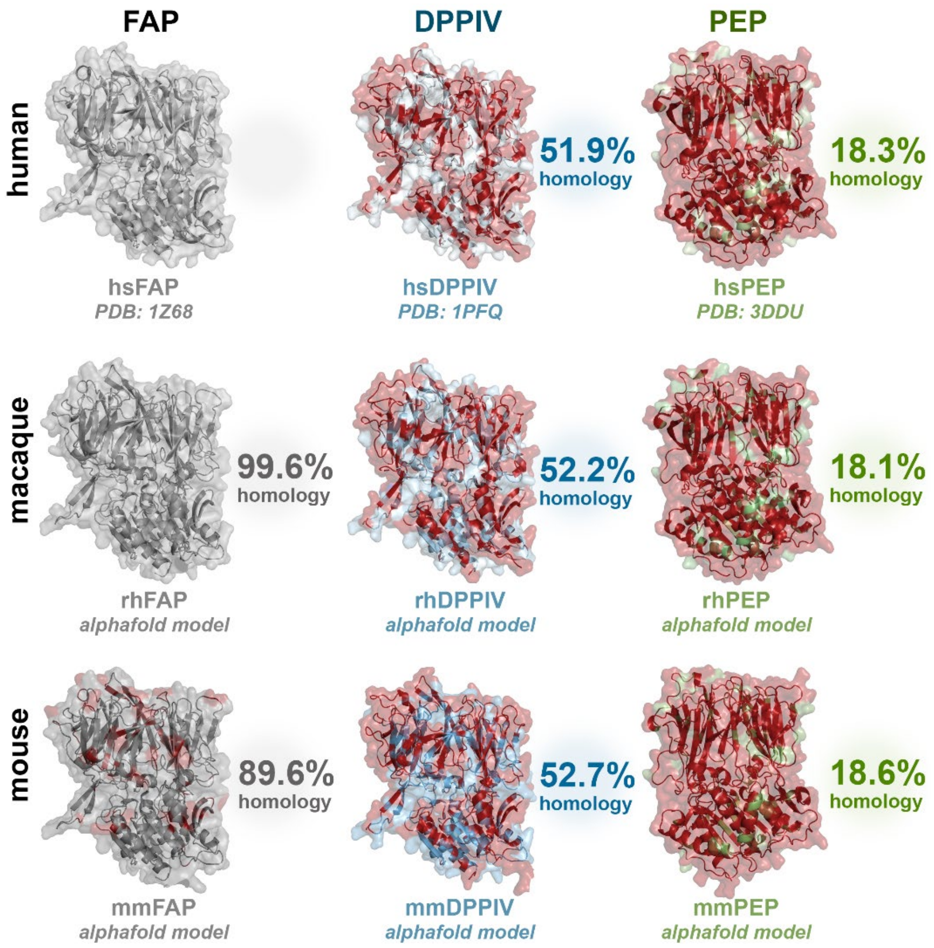

Supplemental Figure 4, Structural and sequence homology of prolyl proteases across species. Structures of fibroblast activation protein (FAP), dipeptidyl peptidase IV (DPPIV), and prolyl oligopeptidase (PEP) are shown for human (top row), rhesus macaque (middle row), and mouse (bottom row). Percent sequence homology to human FAP is shown for each ortholog and paralog. Ribbon and surface depictions highlighted blue or green indicate sequence homology, while red highlights regions with no sequence similarity to human FAP.

Supplemental Figure 5

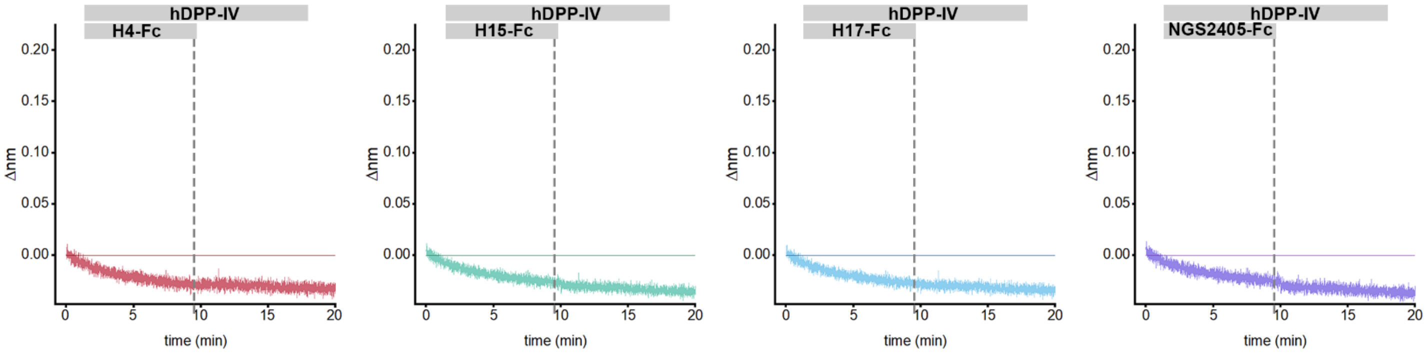

Supplemental Figure 5, anti-FAP VNAR-Fc constructs do not bind to hDPP-IV. Octet biosensors were loaded with biotinylated human DPP-IV and exposed to 2μM concentrations of either H4-Fc (red), H15-Fc (green), H17-Fc (blue), or NGS2405-Fc (purple).

Supplemental Figure 6.

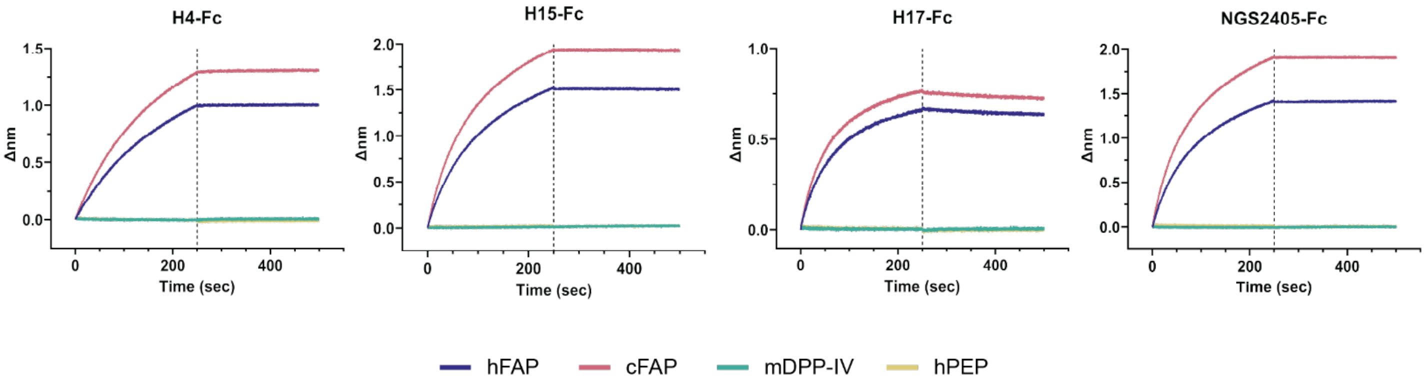

Supplemental Figure 6, anti-FAP VNAR-Fc constructs do not bind to mDPP-IV or hPEP. Octet biosensors were loaded with respective antibody constructs and exposed to 10 µg/mL concentrations of human FAP (hFAP), cynomolgus FAP (cFAP), mouse DPP-IV (mDPP-IV), and human PEP (hPEP).

Supplemental Figure 7

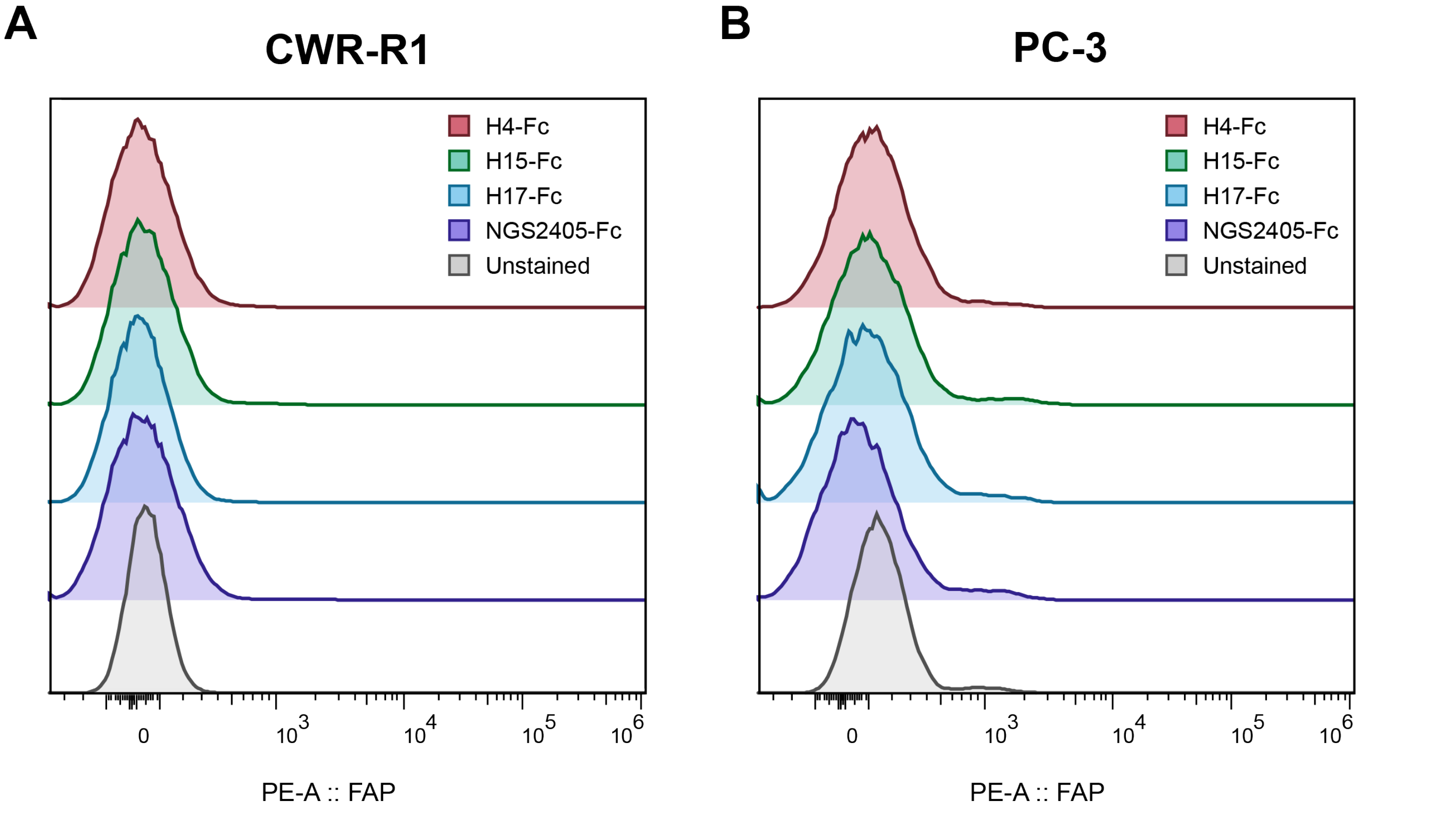

Supplemental Figure 7, anti-FAP VNAR-Fc constructs fail to bind to FAP-negative CWR-R1 and PC-3 prostate cancer cells. Assessing cellular binding of VNAR-Fc constructs to (A) CWR-R1 and (B) PC-3 cell lines by flow cytometry. Cells were stained using a fixed concentration of VNAR-Fc antibodies (100 nM) and detected used a PE labeled anti-human Fc antibody. Samples were compared to an unstained cell control.

Supplemental Figure 8

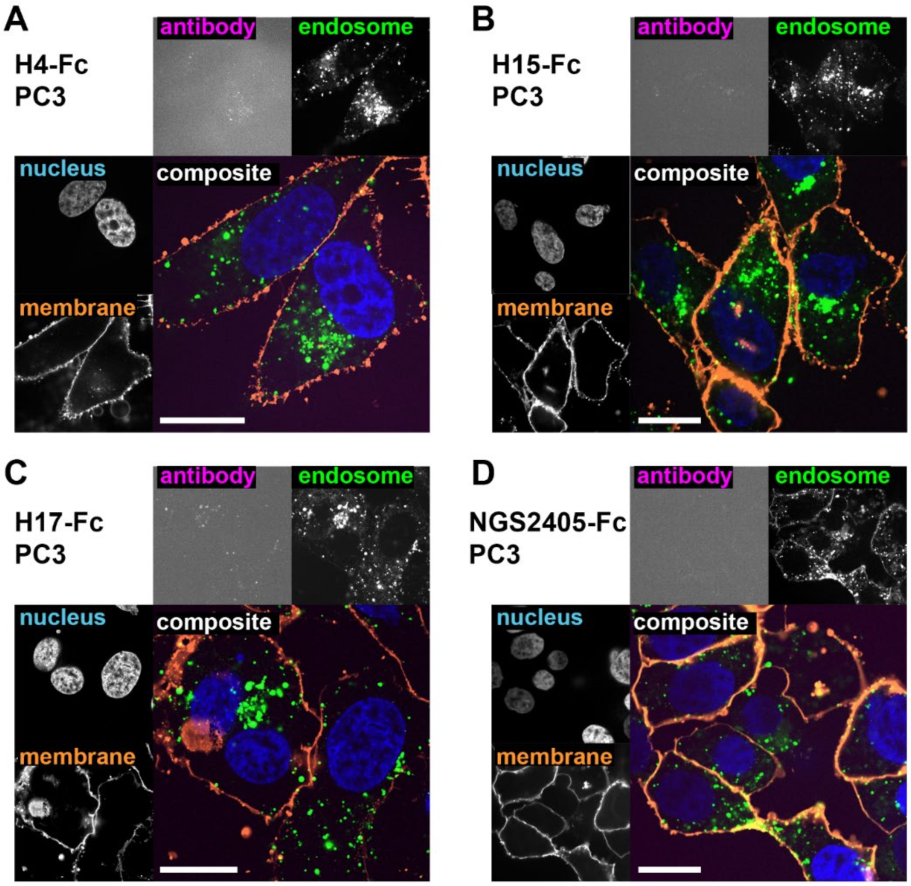

Supplemental Figure 8, anti-FAP VNAR-Fc constructs fail to internalize into FAP-negative PC-3 prostate cancer cells. Confocal microscopy images of PC-3 cells after incubation with H4-Fc-AF647 (A), H15-Fc-AF647 (C), H17-Fc-AF647 (E) or NGS2405-Fc-AF647 (G) for 1hr, using anti-FAP VNAR-Fc-AF647 (10nM) and fluorescein-dextran (50µg/ml). Single-channel images of fluorescein-labeled endosomes, Hoescht 33342-labeled nuclei, and CellBrite 555-labeled membranes are shown. Single-channel images of AF647 fluorescence are shown with high exposure to illustrate the lack of antibody internalization. Merged composite images depicting overlaid colorized fluorescent images are shown, scale bar represents 20µm.

Supplemental Figure 9

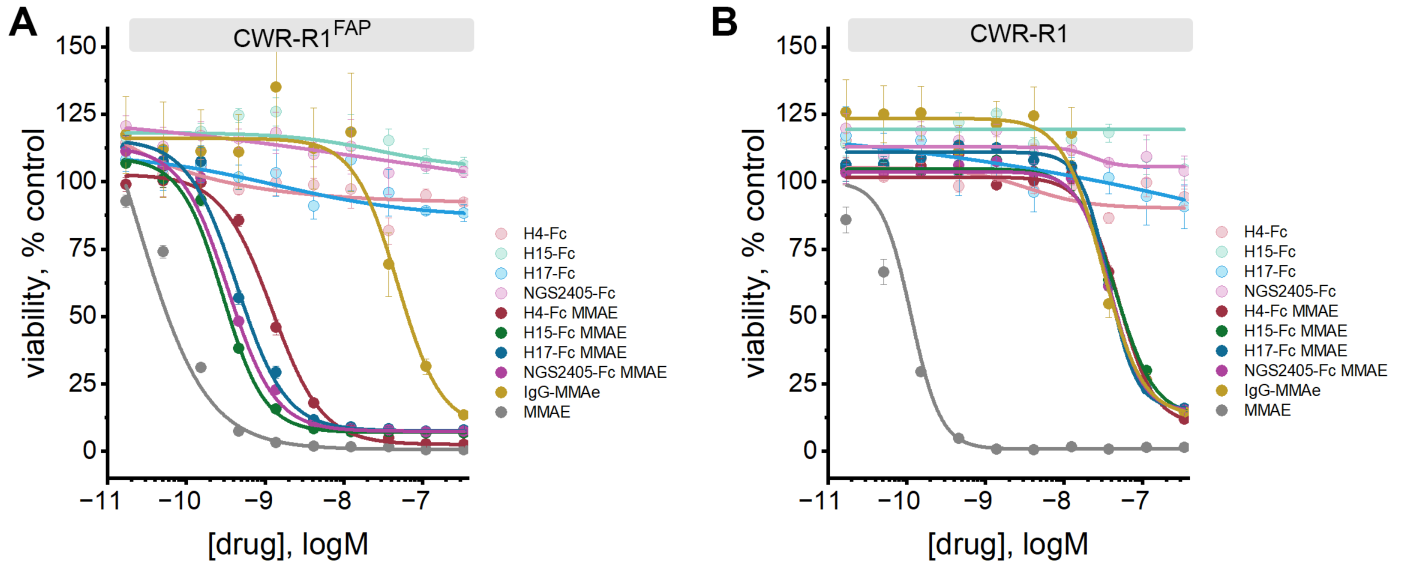

Supplemental Figure 9, Anti-FAP VNAR-Fc-MMAE antibody-drug conjugates dose-dependently kill FAP-expressing cells. Anti-FAP VNAR-Fcs site-specifically conjugated to a monomethyl auristatin E (MMAE) payload were tested for ability to kill CWR-R1^{FAP} (A) and CWR-R1 (B) cells, as detected by measuring the NADPH reductive capacity in cells (CellTiter Blue) after incubation with serially diluted ADCs (300nM-0.03nM). Assays were conducted in parallel with parental unconjugated VNAR-Fc, a non-targeting isotype control VNAR-Fc-MMAE, and free MMAE drug. Data represents mean ± s.e.m. values from n=3 independent experiments.

Supplemental Figure 10

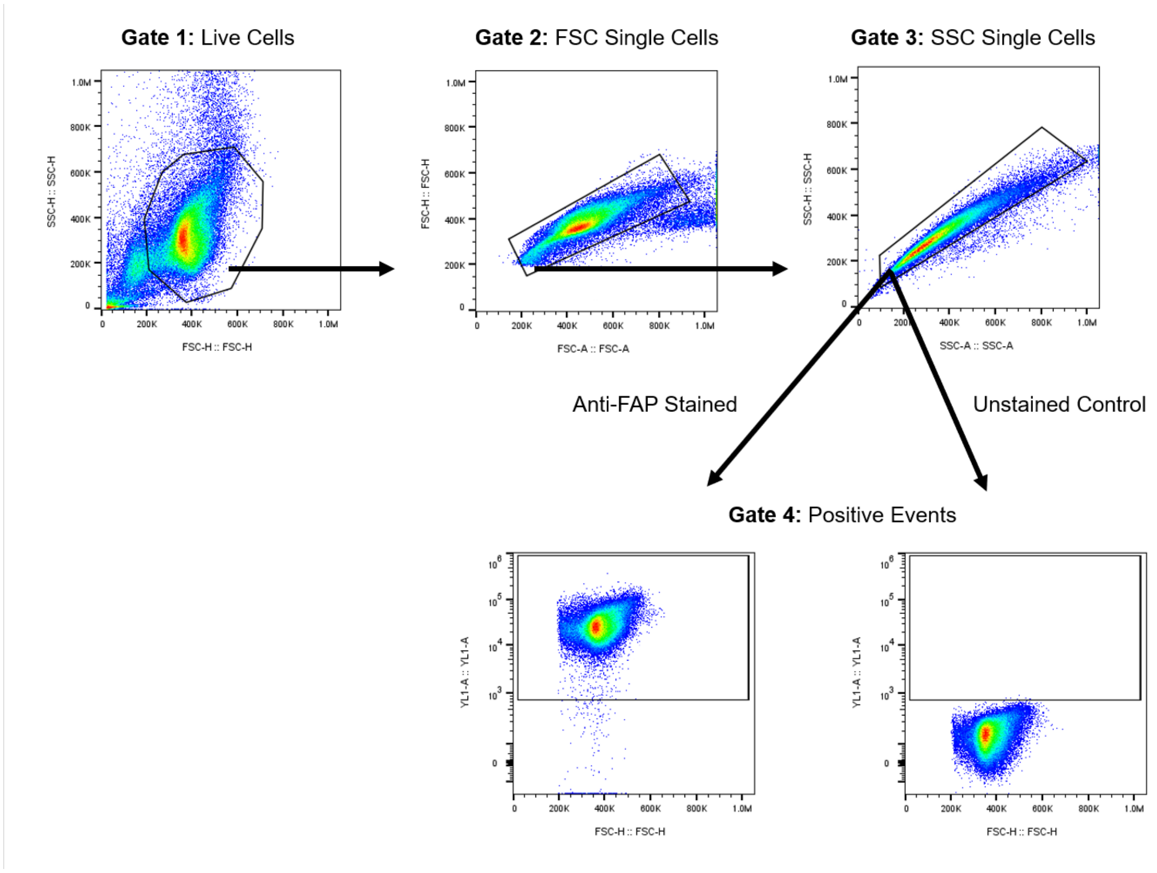

Supplemental Figure 10, Flow cytometry gating strategy for detection of FAP-positive or negative cells. Cells were sequentially gated to identify FAP-positive and negative populations. Gate 1: Live cells were selected based on FSC-H vs. SSC-H to exclude debris. Gate 2: FSC-A vs. FSC-H was used to identify singlets and exclude cell aggregates. Gate 3: SSC-A vs. SSC-H was applied for additional singlet refinement. FAP-positive events were identified based on fluorescence intensity in the YL1-A channel following staining with an anti-FAP primary antibody, detected using an anti-human IgG R-phycoerythrin (PE)-conjugated secondary antibody. An unstained control was used to define background fluorescence and set the gate for FAP-positive events. Dot plots are representative of the gating applied to all samples.

Supplemental Table 1

	hFAP	mFAP	hDPP-IV
**H4-Fc**	1.41E-11 ± 2.7E-12	1.42E-08 ± 3.3E-10	NA
**H15-Fc**	1.28E-09 ± 2.89E-11	NA	NA
**H17-Fc**	5.39E-10 ± 6.2E-12	NA	NA
**NGS2405-Fc**	3.56E-10 ± 8.3E-12	7.95E-08 ± 1.1E-08	NA

Supplemental Table 1. Collated dissociation constants (K_D) of the indicated constructs for human FAP, mouse FAP, or human DPP-IV, as determined by biolayer interferometry.

Supplemental Table 2

	CWR-R1^{FAP}	hPrCSC-44	CWR-R1	PC-3
**H4-Fc**	1.202E-08	1.562E-08	NA	NA
**H15-Fc**	2.469E-09	1.688E-09	NA	NA
**H17-Fc**	2.388E-09	2.207E-09	NA	NA
**NGS2405-Fc**	8.443E-09	6.028E-09	NA	NA

Supplemental Table 2. Collated dissociation constants (K_D) of the indicated constructs for FAP expressing and non-expressing cell lines, as determined by flow cytometry. K_D values in units (M) are derived from logistic fitting of mean fluorescence intensity. NA, K_D not available.
