# Supplementary material for: The characterization of variable new antigen receptors targeting FAP isolated from a novel immunized library
Source: Commun Biol. 2025 Aug 13;8:1210. doi: 10.1038/s42003-025-08610-x (PMC12350670; doi:10.1038/s42003-025-08610-x)
Supplement: Supplementary file 2 — Description of Additional Supplementary Files [file 42003_2025_8610_MOESM2_ESM.docx]

Description of Additional Supplementary Files

**File name:** Supplementary Data 1

**Description:** Source data for the figures and tables.

**File name:** Supplementary Data 2

**Description:** NGS sequencing data.
