# Supplementary material for: The characterization of variable new antigen receptors targeting FAP isolated from a novel immunized library
Source: Commun Biol. 2025 Aug 13;8:1210. doi: 10.1038/s42003-025-08610-x (PMC12350670; doi:10.1038/s42003-025-08610-x)
Supplement: Supplementary file 5 — Reporting Summary [file 42003_2025_8610_MOESM5_ESM.pdf]

nature portfolio

Corresponding author(s): Aaron LeBeau

Last updated by author(s): Jun 16, 2025

Reporting Summary

Nature Portfolio wishes to improve the reproducibility of the work that we publish. This form provides structure for consistency and transparency in reporting. For further information on Nature Portfolio policies, see our Editorial Policies and the Editorial Policy Checklist.

Statistics

For all statistical analyses, confirm that the following items are present in the figure legend, table legend, main text, or Methods section.

n/a	Confirmed	
☐	☒	The exact sample size (*n*) for each experimental group/condition, given as a discrete number and unit of measurement
☐	☒	A statement on whether measurements were taken from distinct samples or whether the same sample was measured repeatedly
☐	☒	The statistical test(s) used AND whether they are one- or two-sided<br>*Only common tests should be described solely by name; describe more complex techniques in the Methods section.*
☐	☒	A description of all covariates tested
☐	☒	A description of any assumptions or corrections, such as tests of normality and adjustment for multiple comparisons
☐	☒	A full description of the statistical parameters including central tendency (e.g. means) or other basic estimates (e.g. regression coefficient) AND variation (e.g. standard deviation) or associated estimates of uncertainty (e.g. confidence intervals)
☒	☐	For null hypothesis testing, the test statistic (e.g. F, t, r) with confidence intervals, effect sizes, degrees of freedom and P value noted<br>*Give P values as exact values whenever suitable.*
☒	☐	For Bayesian analysis, information on the choice of priors and Markov chain Monte Carlo settings
☐	☒	For hierarchical and complex designs, identification of the appropriate level for tests and full reporting of outcomes
☐	☒	Estimates of effect sizes (e.g. Cohen's *d*, Pearson's *r*), indicating how they were calculated

Our web collection on statistics for biologists contains articles on many of the points above.

Software and code

Policy information about availability of computer code

Data collection	Biolayer interferometry data was collected using Forte Bio evaluation software on an Octet Red96E. Flow cytometry data was acquired on an Attune™ NxT flow cytometer (Thermo Fisher Scientific). Internalization and cell killing assays were performed using an Incucyte with the provided software. PET data was acquired on a Siemens Inveon microPET/CT with data processed using Amira software.
Data analysis	Data was processed in MicrosoftExcel as described in the Methods section, concentration-response curves and IC50 values were generated using Origin Lab 2021b. BLI data was processed in GraphPad Prismv9.0. Flow cytometry data was analyzed using Flo Jo software.

For manuscripts utilizing custom algorithms or software that are central to the research but not yet described in published literature, software must be made available to editors and reviewers. We strongly encourage code deposition in a community repository (e.g. GitHub). See the Nature Portfolio guidelines for submitting code & software for further information.

Data

Policy information about availability of data

All manuscripts must include a data availability statement. This statement should provide the following information, where applicable:

- Accession codes, unique identifiers, or web links for publicly available datasets
- A description of any restrictions on data availability
- For clinical datasets or third party data, please ensure that the statement adheres to our policy

All plasmids were sourced as indicted in the Methods. No data availability restrictions will be made, all source data has been uploaded.

Research involving human participants, their data, or biological material

Policy information about studies with human participants or human data. See also policy information about sex, gender (identity/presentation), and sexual orientation and race, ethnicity and racism.

Reporting on sex and gender	n/a
Reporting on race, ethnicity, or other socially relevant groupings	n/a
Population characteristics	n/a
Recruitment	n/a
Ethics oversight	n/a

Note that full information on the approval of the study protocol must also be provided in the manuscript.

Field-specific reporting

Please select the one below that is the best fit for your research. If you are not sure, read the appropriate sections before making your selection.

☒ Life sciences ☐ Behavioural & social sciences ☐ Ecological, evolutionary & environmental sciences

For a reference copy of the document with all sections, see nature.com/documents/nr-reporting-summary-flat.pdf

Life sciences study design

All studies must disclose on these points even when the disclosure is negative.

Sample size	Samples sizes are described in the Methods section along with statistical analysis methods used.
Data exclusions	No data exclusions were made.
Replication	Biological experiments performed with at least three replicates. All attempts at replication were successful, no data was excluded from analyses.
Randomization	n/a
Blinding	n/a

Reporting for specific materials, systems and methods

We require information from authors about some types of materials, experimental systems and methods used in many studies. Here, indicate whether each material, system or method listed is relevant to your study. If you are not sure if a list item applies to your research, read the appropriate section before selecting a response.

Materials & experimental systems

n/a	Involved in the study	
☐	☒	Antibodies
☐	☒	Eukaryotic cell lines
☒	☐	Palaeontology and archaeology
☐	☒	Animals and other organisms
☒	☐	Clinical data
☒	☐	Dual use research of concern
☒	☐	Plants

Methods

n/a	Involved in the study	
☒	☐	ChIP-seq
☐	☒	Flow cytometry
☒	☐	MRI-based neuroimaging

Antibodies

Antibodies used	PE labeled goat anti-human IgG Fc (eBioscience, Invitrogen), anti-HA-tag monoclonal antibody conjugated to peroxidase (12-013-819-001, Sigma-Aldrich, St. Louis, Mo.)

Validation	Novel antibodies reported in this manuscript are validated in this work

Eukaryotic cell lines

Policy information about cell lines and Sex and Gender in Research

Cell line source(s)	American Type Culture Collection (ATCC)
Authentication	All cell lines have been authenticated using short-tandem repeat profiling.
Mycoplasma contamination	Cells are routinely monitored for mycoplasma contamination using a mycoplasma detection kit.
Commonly misidentified lines (See ICLAC register)	n/a

Animals and other research organisms

Policy information about studies involving animals; ARRIVE guidelines recommended for reporting animal research, and Sex and Gender in Research

Laboratory animals	Athymic Nude-Foxn1nu mice (Envigo) and nurse sharks
Wild animals	n/a
Reporting on sex	n/a
Field-collected samples	n/a
Ethics oversight	All animal studies were approved by the University of Wisconsin Institutional Animal Care and Use Committee.

Note that full information on the approval of the study protocol must also be provided in the manuscript.

Plants

Seed stocks	n/a
Novel plant genotypes	n/a
Authentication	n/a

Flow Cytometry

Plots

Confirm that:

☒ The axis labels state the marker and fluorochrome used (e.g. CD4-FITC).

☒ The axis scales are clearly visible. Include numbers along axes only for bottom left plot of group (a 'group' is an analysis of identical markers).

☒ All plots are contour plots with outliers or pseudocolor plots.

☒ A numerical value for number of cells or percentage (with statistics) is provided.

Methodology

Sample preparation	For flow cytometry studies, CWR-R1, CWR-R1FAP, hPrCSC-44, and PC3 cell lines were harvested and suspended in flow cytometry staining buffer (eBioscience, Invitrogen) at a final concentration of 1 x 106 cells/mL. Cells were then incubated with various concentrations of VNAR-Fcs for 1 hour on ice. After incubation, cells were centrifuged at 400g for 4 minutes and washed with staining buffer thrice. Pelleted cells were then resuspended in staining buffer containing PE labeled goat anti-human IgG Fc (5 µg/mL) (eBioscience, Invitrogen) for 45 min on ice. Cells were washed and resuspended in staining buffer thrice.
Instrument	Samples were analyzed on a Attune Flow Cytometer (ThermoFisher)

Software	fluorescence intensity analysis and histograms generation were done using FlowJo software (Tree Star, Inc.).
Cell population abundance	The cells were acquire from ATCC and had high viability >95% when the experiments were performed. These cells were verified by STR analysis and were negative for mycoplasm. For the analysis, the cells were gated to omit any potential cellular debris resulting from the experiment.
Gating strategy	Cells were gated based onon FCS-A vsvs SSC-A toto isolate live cell populations. Single cell populations were isolated byby gating FCS-A vs. FCS-H as well as SSC-A vs SSC-H. Single cell population were plotted on an FCS-H vs R1-A axis to determine MFI.

☒ Tick this box to confirm that a figure exemplifying the gating strategy is provided in the Supplementary Information.
